# Supplementary material for: Iodide manipulation using zinc additives for efficient perovskite solar minimodules
Source: Nat Commun. 2024 Feb 14;15:1355. doi: 10.1038/s41467-024-45649-6 (PMC10867015; doi:10.1038/s41467-024-45649-6)
Supplement: Supplementary file 1 — Supplementary Information [file 41467_2024_45649_MOESM1_ESM.pdf]

Supplementary Information

For

Iodide Manipulation Using Zinc Additives for Efficient Perovskite Solar Minimodules

Md Aslam Uddin,[1] Prem Jyoti Singh Rana,[1] Zhenyi Ni,[1] Guang Yang,[1] Mingze Li,[1] Mengru Wang,[1] Hangyu Gu,[1] Hengkai Zhang,[1] Benjia Dak Dou,[2] and Jinsong Huang*[1,3]

[1]Department of Applied Physical Sciences, University of North Carolina at Chapel Hill, Chapel Hill, NC 27599, USA

[2]CubicPV Inc., Bedford, MA 01730, USA

[3]Department of Chemistry, University of North Carolina at Chapel Hill, Chapel Hill, NC 27599, USA

*E-mail: jhuang@unc.edu

Table of Contents

This file includes:

Supplementary Figures 1-10

Supplementary Tables 1-4

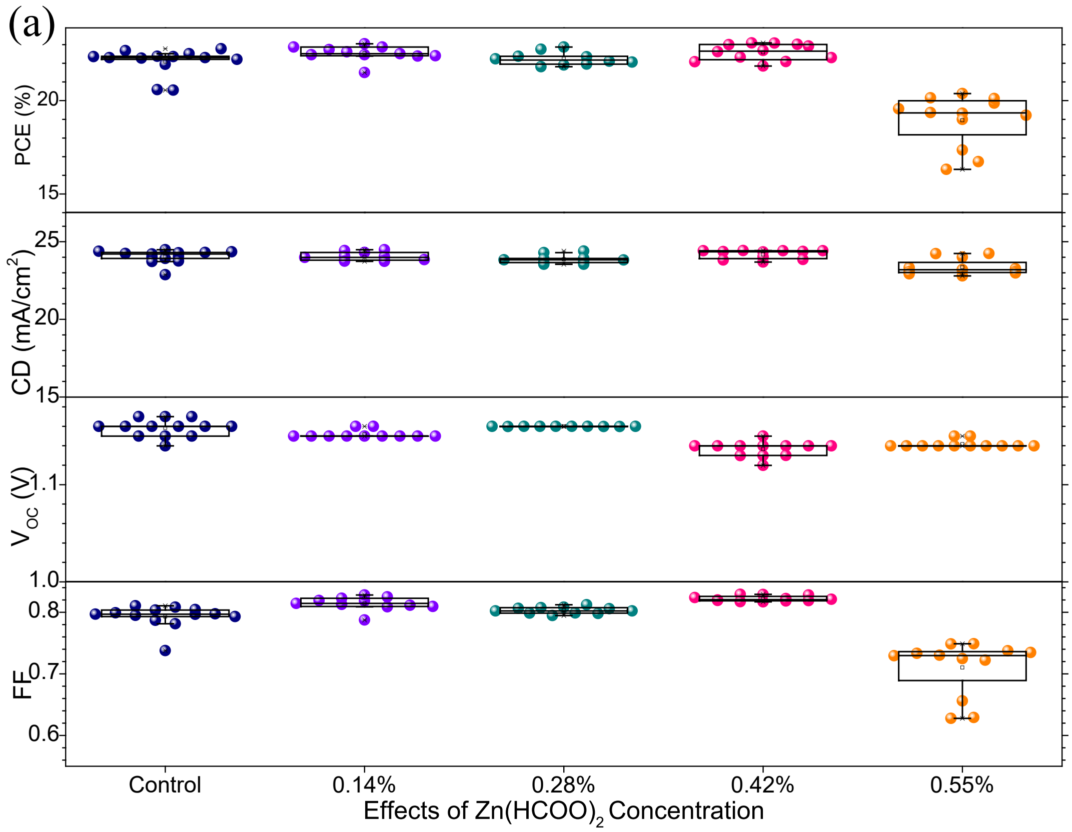
(a)
PCE (%)
20
15
CD (mA/cm^2)
25
20
15
V_{OC} (V)
1.1
1.0
FF
0.8
0.7
0.6
Control
0.14%
0.28%
0.42%
0.55%
Effects of $Zn(HCOO)_2$ Concentration

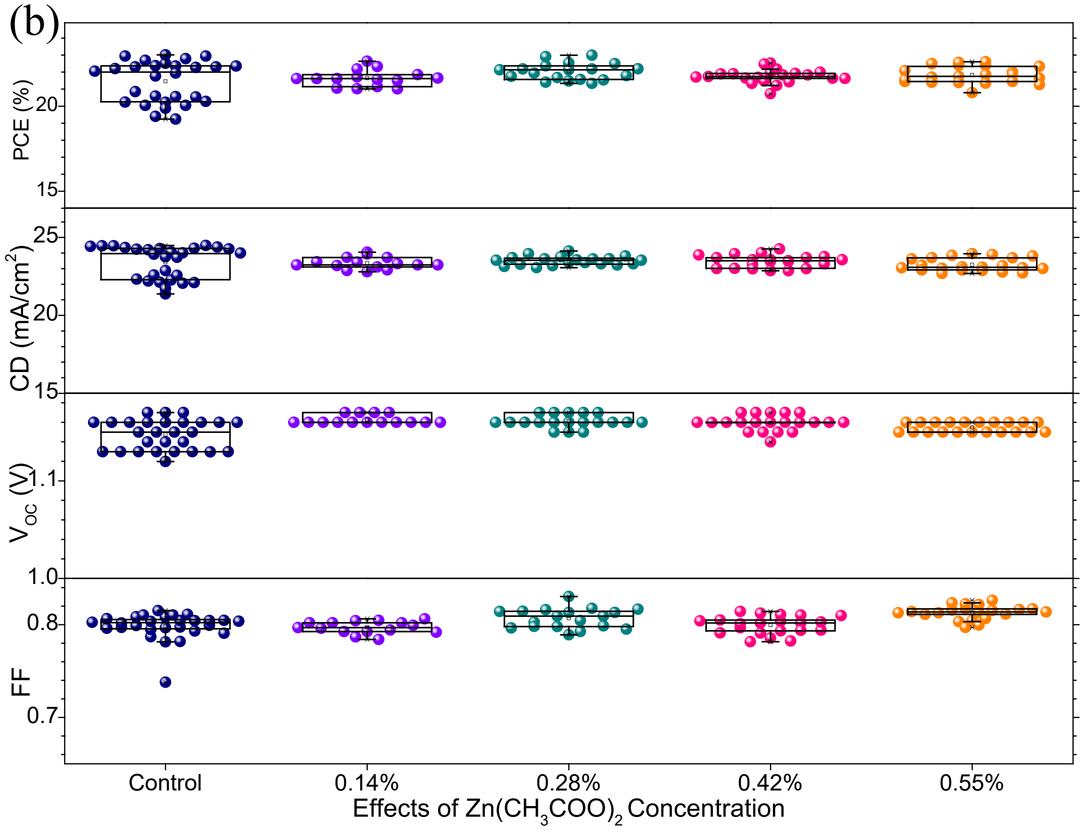
(b)
PCE (%)
20
15
CD (mA/cm^2)
25
20
15
V_{OC} (V)
1.1
1.0
FF
0.8
0.7
Control
0.14%
0.28%
0.42%
0.55%
Effects of $Zn(CH_3COO)_2$ Concentration

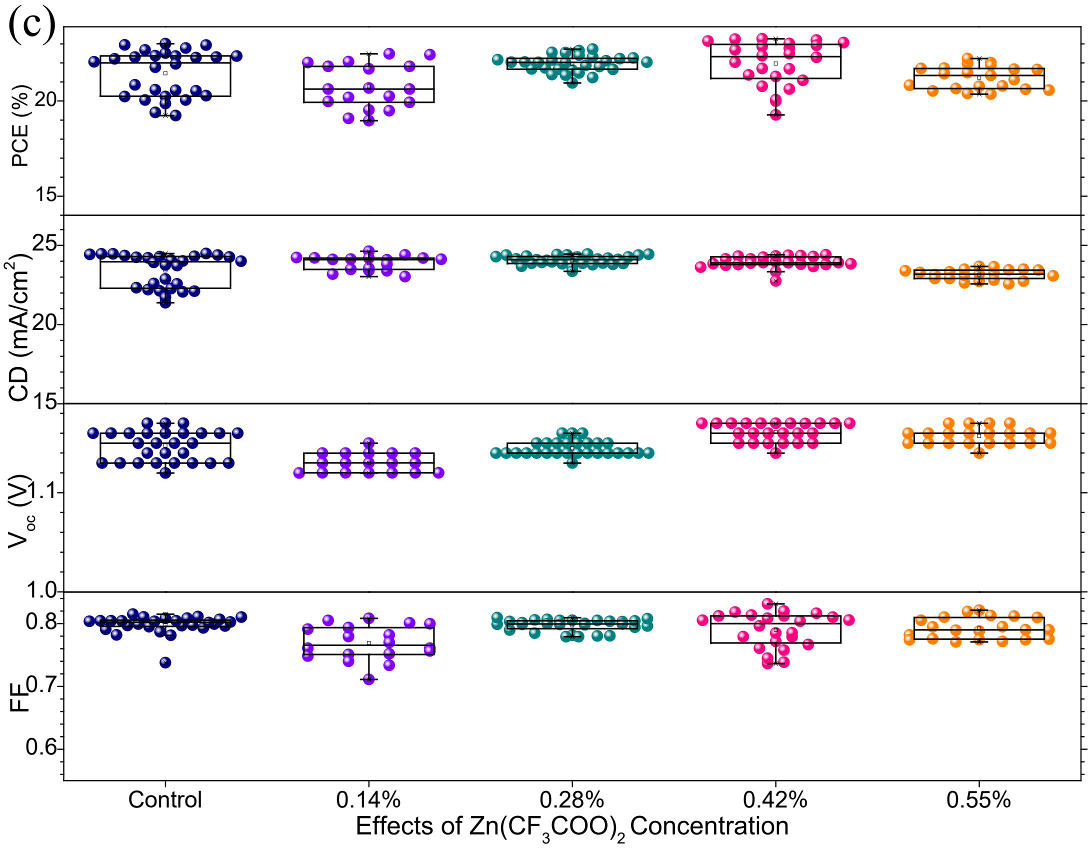
(c)
PCE (%)
20
15
CD (mA/cm2)
25
20
15
Voc (V)
1.1
1.0
FF
0.8
0.7
0.6
Control
0.14%
0.28%
0.42%
0.55%
Effects of Zn(CF3COO)2 Concentration

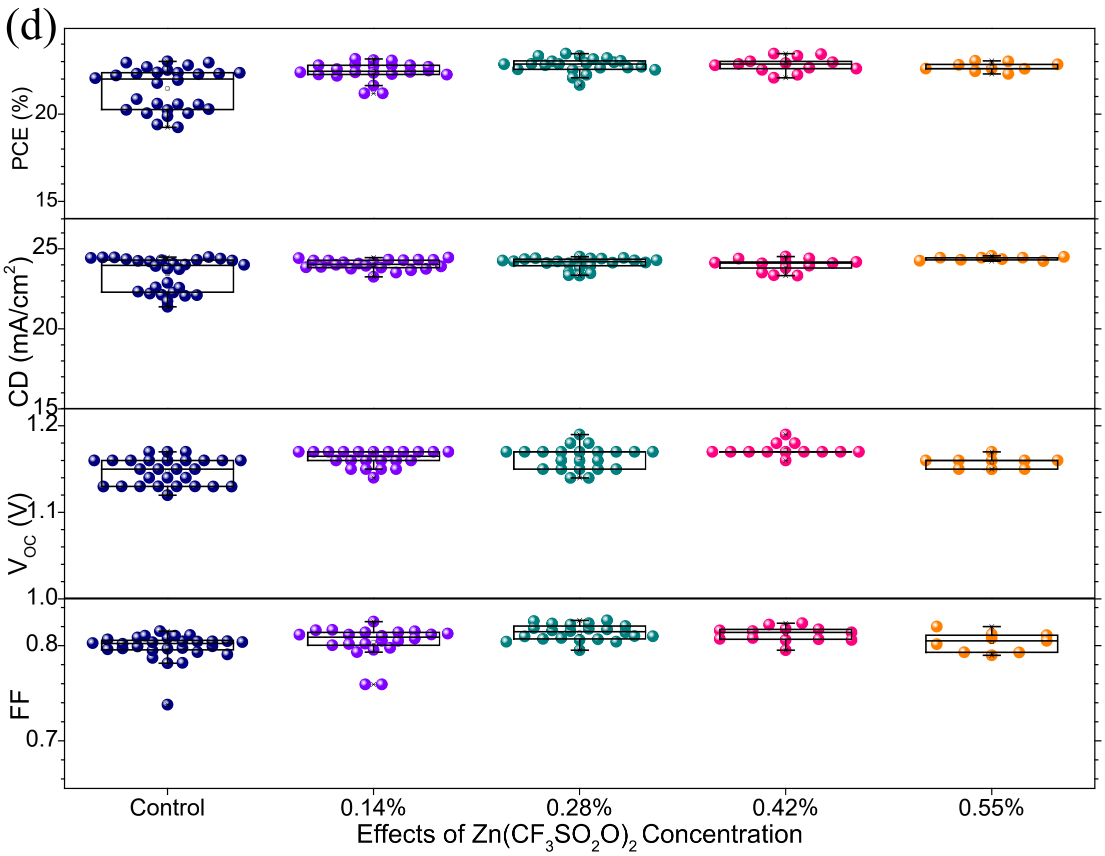

Fig.S1. Distribution of performance of devices (20-25 devices for each concentration) fabricated with each additive- **(a)** $Zn(OOCH)_2$, **(b)** $Zn(OOCCH_3)_2$, **(c)** $Zn(OOCCF_3)_2$, and **(d)**$Zn(OO_2SCF_3)_2$ with varying concentration. Each dot indicates an individual PV cell with an active area of 0.08 cm^2.

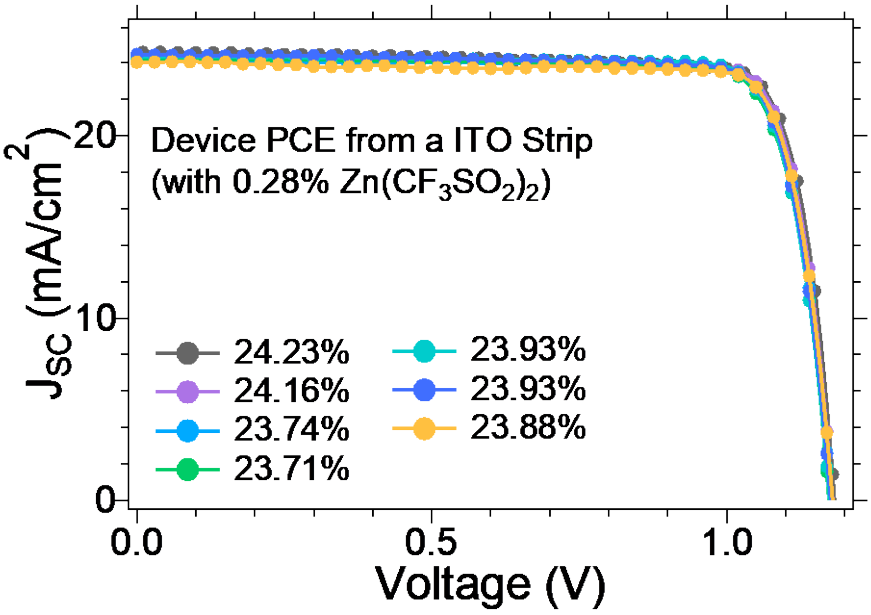

Fig.S2. J-V curves show the effects of optimized concentration of 0.28% $Zn(OOSCF_3)_2$ on the reproducibility of small devices from a single strip of ITOs with an active area of 0.08 cm^2.

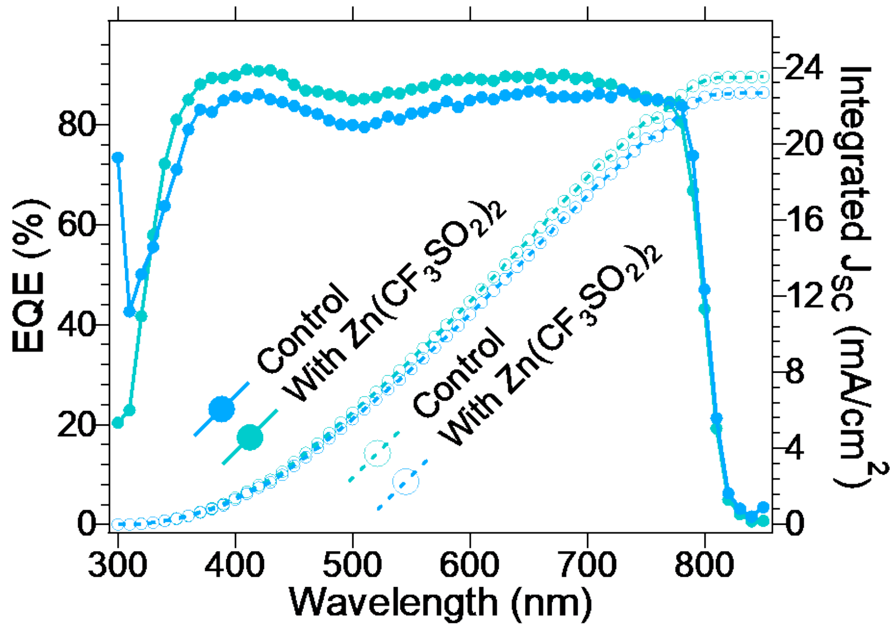

Fig.S3. Comparison of *J-V* characteristics and external quantum efficiency (EQE) spectra and integrated J_{sc} of a control device and a target small device with 0.28% Zn(OOSCF$_3$)$_2$ added perovskites..

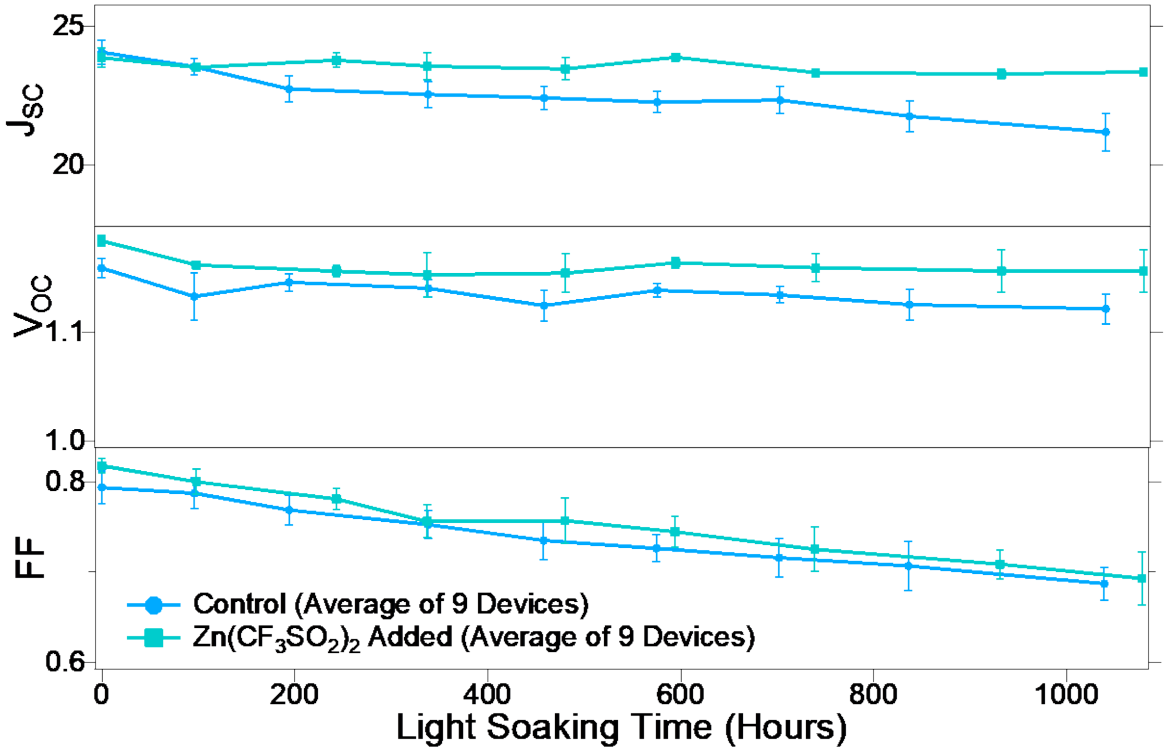

Fig.S4. Time-dependent stability of control and target small devices with added 0.42% $Zn(OOSCF_3)_2$ at V_{OC} condition under one sun of LED light at 55±5 ºC and 50±20% RH.

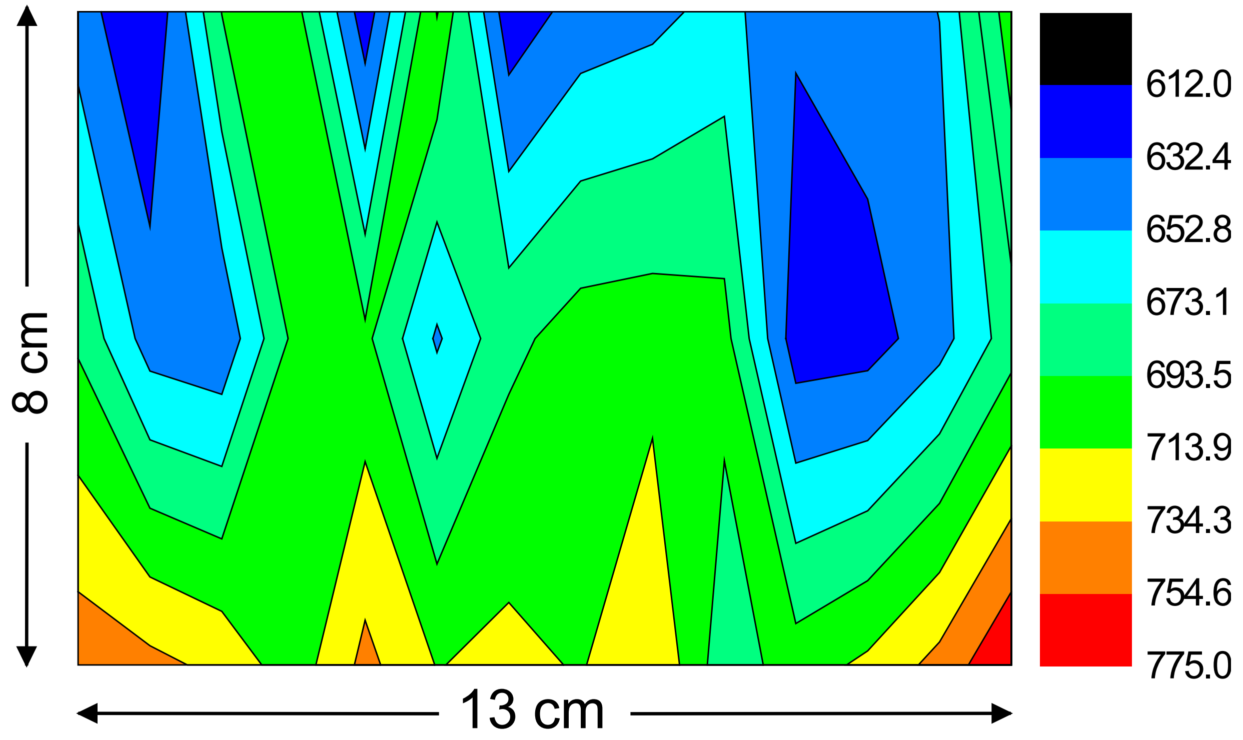

Fig.S5. A contour diagram of the thickness of a bladed $FA_{0.3}MA_{0.7}PbI_3$ perovskite film on a large area substrate (~112 cm^2) at different locations was measured using a Dektak XT profiler.

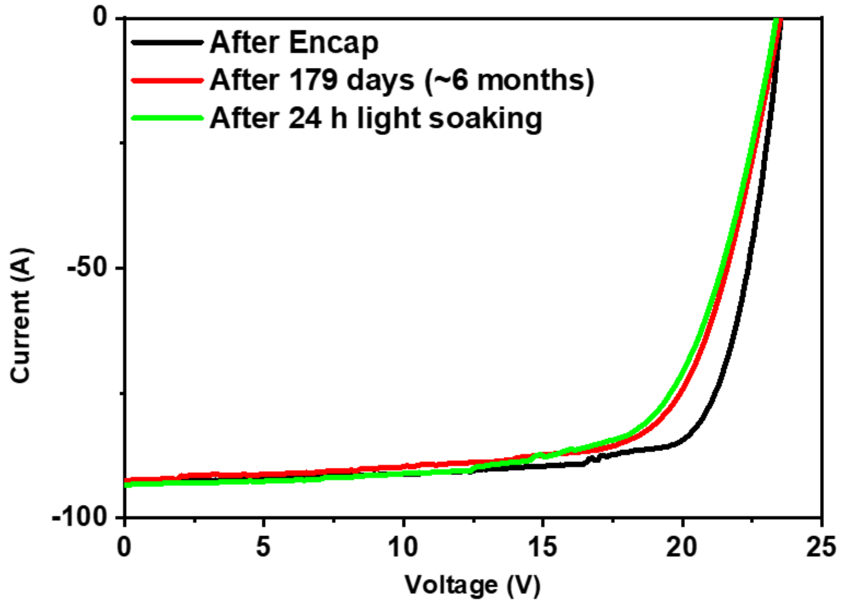

Fig.S6. *J*-*V* scans show the stability of an epoxy-encapsulated minimodule with an aperture area of 79.67 cm^2.

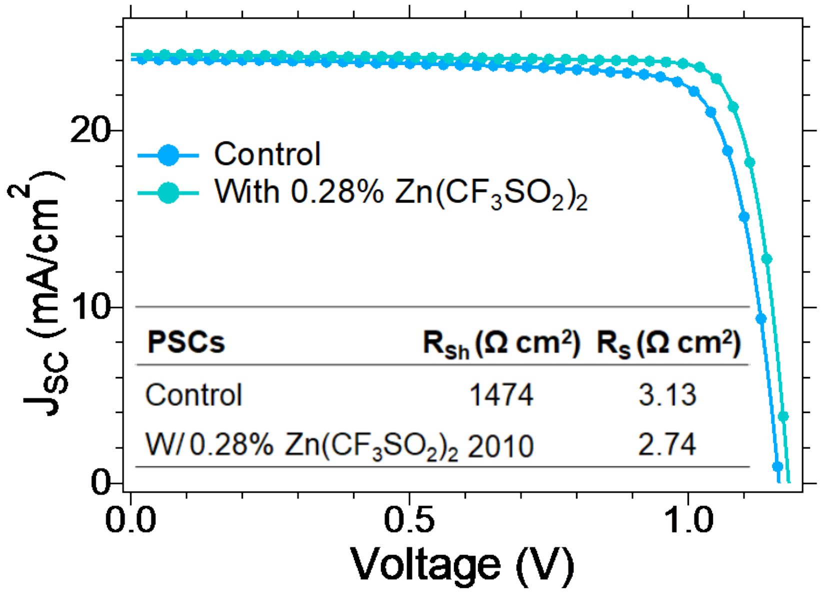

Fig.S7. *J*-*V* curves show the effects of Zn concentrations $(OOSCF_3)_2$ on the series and shunt resistance.

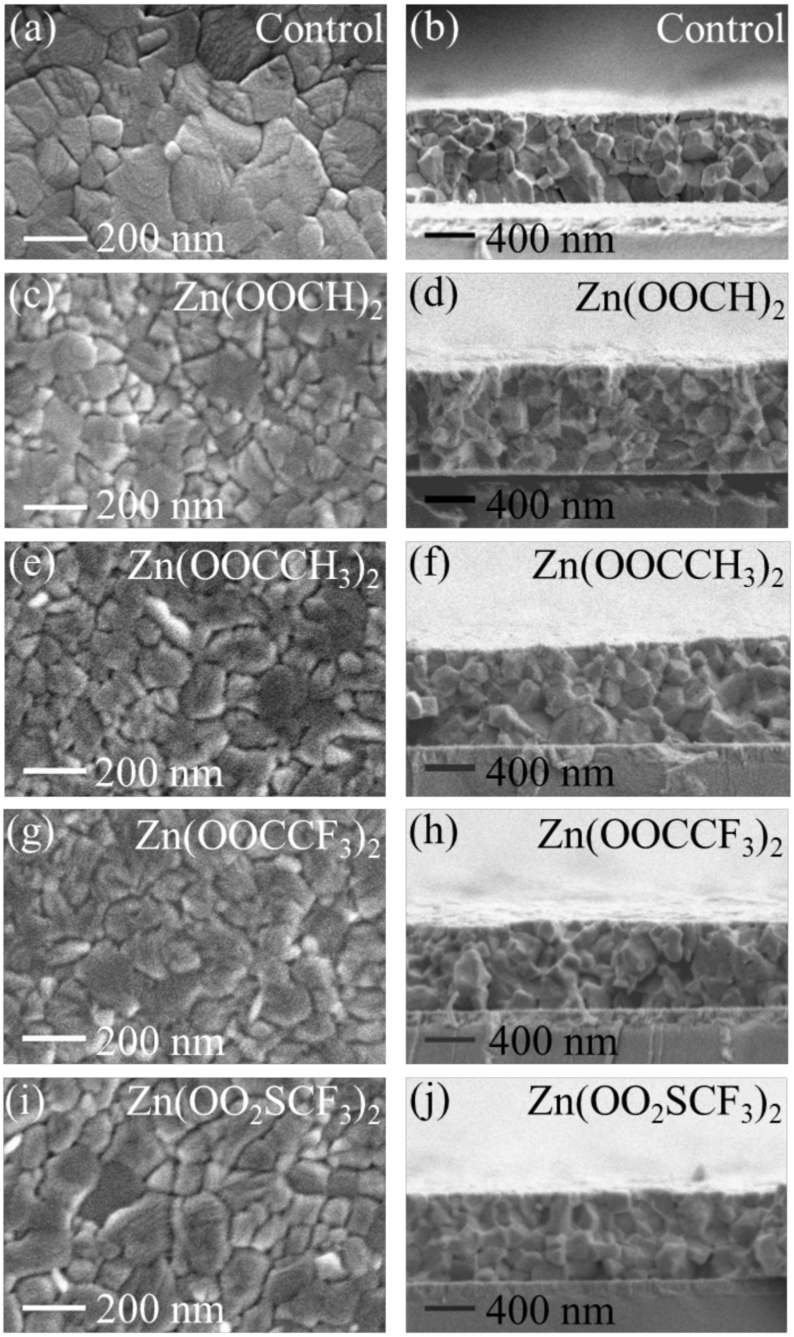

Fig.S8. Comparison of the top surface and cross-section SEM images of **(a, b)** control, **(c, d)** $Zn(OOCH)_2$, **(e, f)** $Zn(OOCCH_3)_2$, **(g, h)** $Zn(OOCCF_3)_2$, and **(i, j)** $Zn(OO_2SCF_3)_2$ added perovskite films.

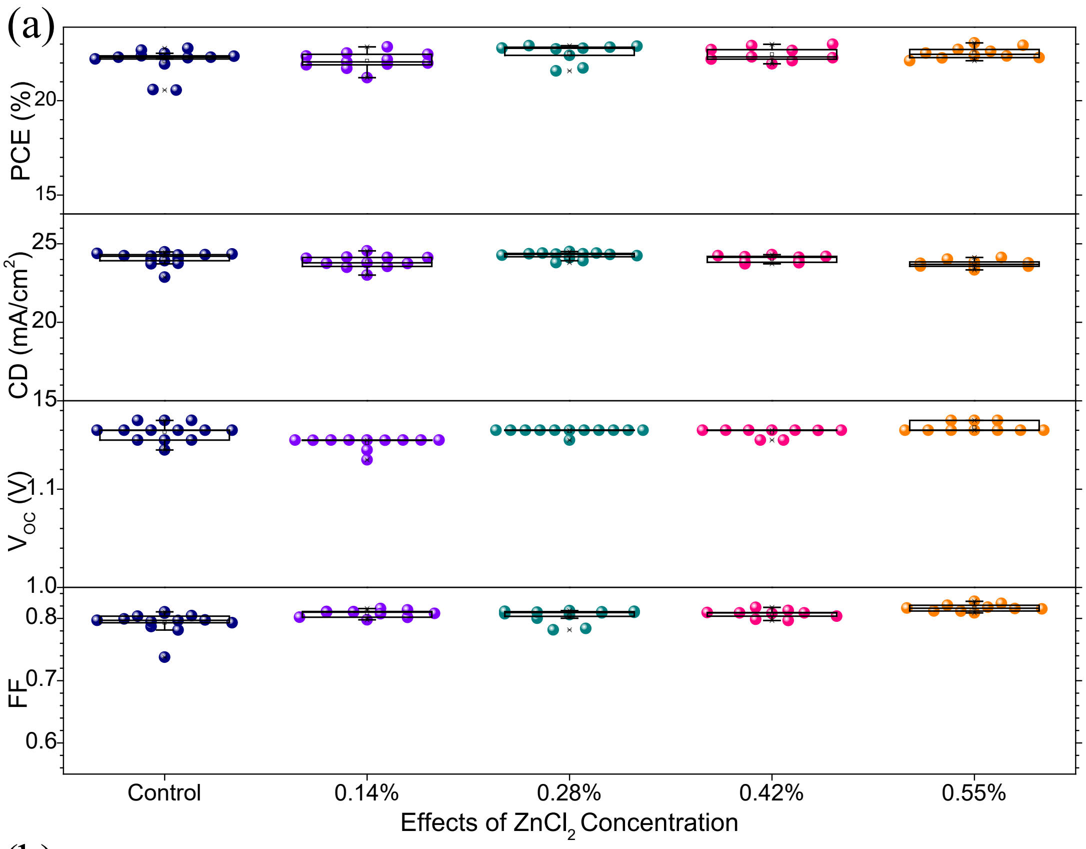
(a)
PCE (%)
20
15
CD (mA/cm^2)
25
20
15
V_{OC} (V)
1.1
1.0
FF
0.8
0.7
0.6
Control
0.14%
0.28%
0.42%
0.55%
Effects of $ZnCl_2$ Concentration

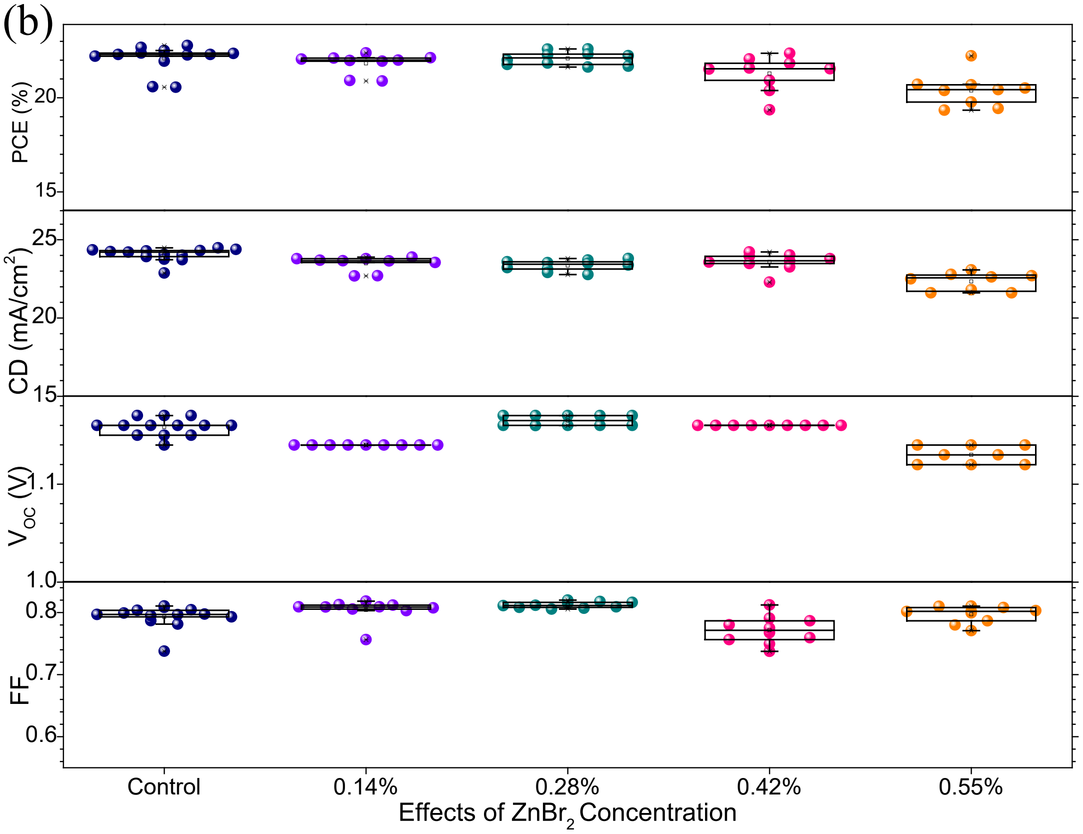
(b)
PCE (%)
20
15
CD (mA/cm^2)
25
20
15
V_{OC} (V)
1.1
1.0
FF
0.8
0.7
0.6
Control
0.14%
0.28%
0.42%
0.55%
Effects of $ZnBr_2$ Concentration

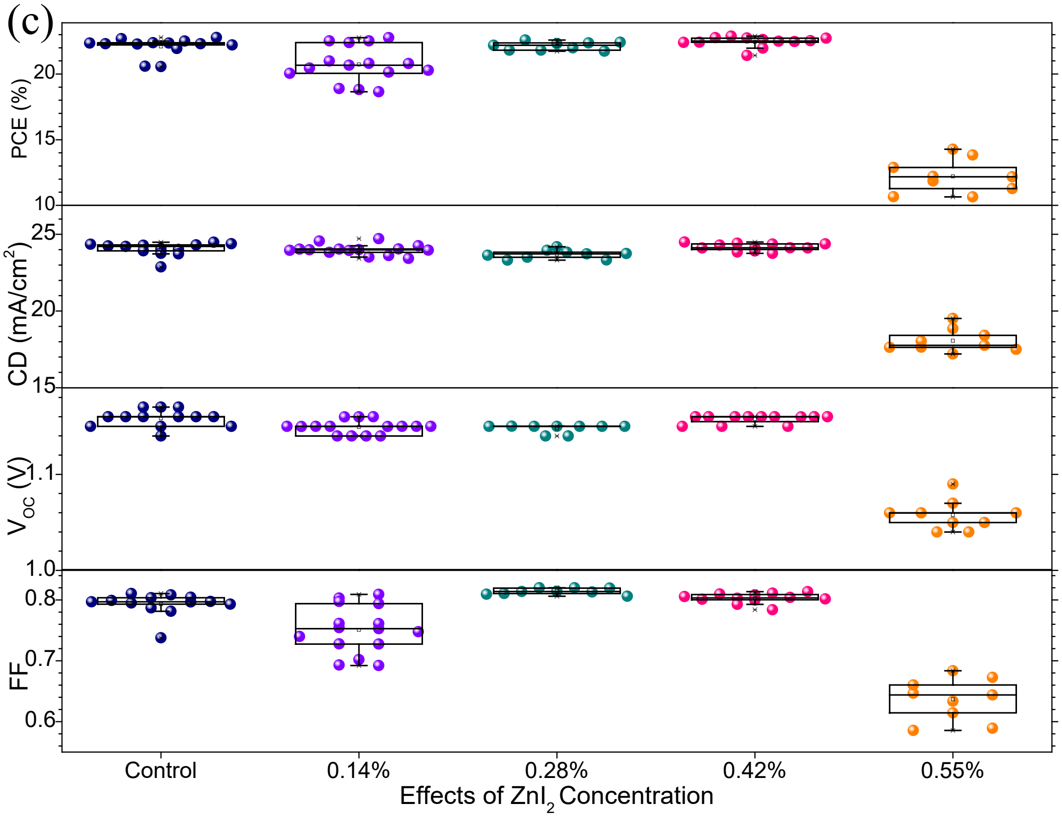

Fig.S9. Performance distribution of the devices (10-15 devices for each concentration) fabricated with each additive- **(a)** $ZnCl_2$, **(b)** $ZnBr_2$, and **(c)** ZnI_2 with varying concentrations. Each dot indicates an individual PV cell with an active area of 0.08 cm^2.

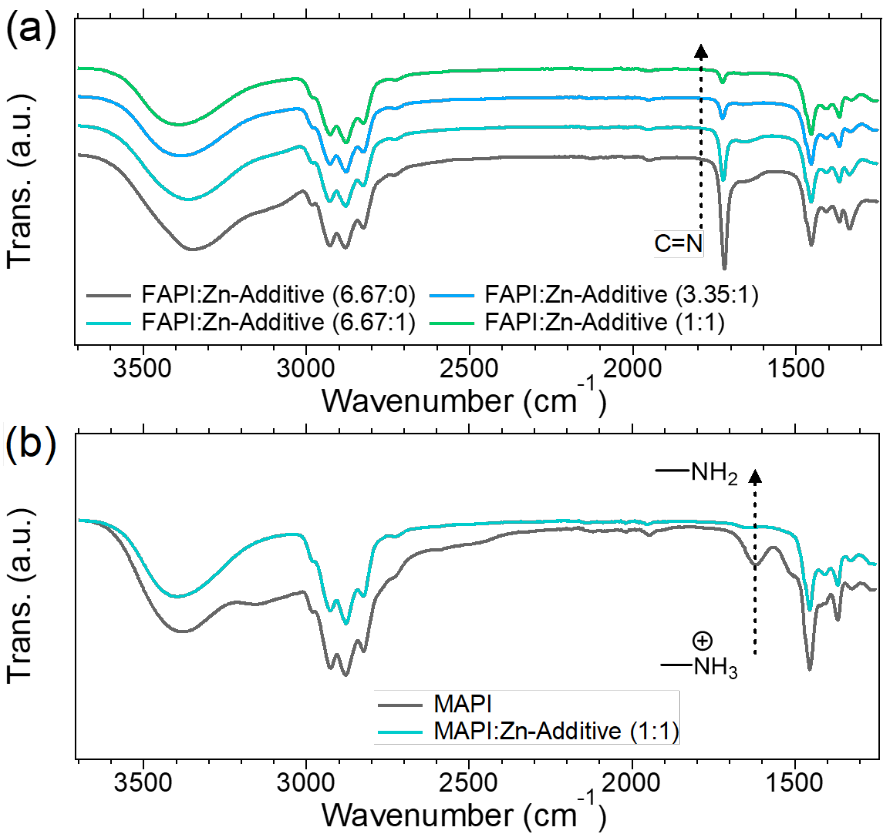

Fig.S10. (**a**) FTIR spectra of $FAPbI_3$ (FAPI) and FAPI: $Zn(OOSCF_3)_2$ in different molar ratios in 2-ME and **(b)** FTIR spectra of $MAPbI_3$ (MAPI) and MAPI: $Zn(OOSCF_3)_2$ in a molar ratio of 1:1 in 2-ME.

Table S1: Photovoltaic performance of small devices with 0.28% $Zn(OOSCF_3)_2$.

Additives	Device Area (cm^2)	Jsc (mA/cm2)	Voc	FF	Eff
0.28% $Zn(O_2SCF_3)_2$	0.08	24.33598	1.179858	0.828257	23.78173
		24.50922	1.179892	0.801899	23.1895
		24.44508	1.179862	0.804493	23.20303
		24.5644	1.179876	0.824175	23.88703
		24.50223	1.17991	0.815635	23.58037
		24.50223	1.17991	0.815635	23.58037
		24.43199	1.179885	0.803668	23.16728
		24.15774	1.179891	0.829745	23.65063
		24.35609	1.189923	0.812456	23.54651
		24.44542	1.179892	0.825423	23.80763
		24.51688	1.179897	0.816514	23.61962
		24.4224	1.179892	0.808552	23.29908
		24.33773	1.179884	0.822666	23.62342
		24.38443	1.179891	0.813592	23.40784
		24.49848	1.179893	0.797537	23.05326
		24.44608	1.179884	0.826348	23.83481
		24.53769	1.179882	0.81944	23.72409
		24.39599	1.179892	0.808421	23.27011
		24.4013	1.179882	0.826612	23.79871

		24.44875	1.179911	0.807999	23.30863
		24.28358	1.179902	0.826764	23.68865
		24.3796	1.179718	0.813141	23.3868
		24.3796	1.179718	0.813141	23.3868
		23.79775	1.179878	0.849664	23.85724
		24.31349	1.179746	0.842372	24.16239
		24.187	1.179888	0.831894	23.74056
		24.20189	1.169877	0.837304	23.70679
		24.32723	1.169889	0.840944	23.93341
		24.46081	1.179857	0.82922	23.9315
		24.01881	1.179766	0.842994	23.88755

Table S2: Photovoltaic performance of submodules with $Zn(OOSCF_3)_2$ additive.

Additive	No. of Modules	Aperture area (cm2)	No. of Sub-cells	Jsc (mA/cm2)	Voc (V)	FF	PCE (%)
	1	78	20	1.08264	23.3003	0.77715	19.6041
	2		20	1.08264	23.3003	0.77715	19.6041
	3		20	1.08264	23.3003	0.77715	19.6041
	4		20	1.08264	23.3003	0.77715	19.6041
	5		20	1.08264	23.3003	0.77715	19.6041
	6		20	1.08264	23.3003	0.77715	19.6041
	7		20	1.08264	23.3003	0.77715	19.6041
	8		20	1.10715	22.9665	0.76613	19.4807
	9		20	1.11456	23.5131	0.76437	20.0317
	10		20	1.0634	23.5134	0.76195	19.0519
	11		20	1.11671	23.4768	0.76411	20.0326
	12		19	1.10883	22.2132	0.77425	19.0702
	13		20	1.1199	23.1697	0.77036	19.8299
	14		20	1.1135	23.1823	0.7401	19.1047
	15		20	1.1142	23.2194	0.76891	19.8925
	16		20	1.04673	23.1699	0.78622	19.068
	17		20	1.11354	23.3574	0.75275	19.5788
	18		20	1.10539	23.3654	0.779	20.1197

	19		20	1.10688	23.2706	0.7543	19.429
	20		20	1.07216	23.1846	0.77122	19.1705
	21		19	1.11709	22.2153	0.7751	19.2353
	22		19	1.0881	22.2862	0.75397	18.2834
	23		20	1.10929	23.3026	0.76142	19.6821
	24		20	1.07296	23.4775	0.80124	20.1835
	25		20	1.03146	23.4108	0.74722	18.0433
	26		20	1.10617	22.9792	0.77183	19.619
	27	84	20	1.09051	23.50132	0.75342	19.30886
	28		20	1.0792	23.50383	0.79386	20.13632
	29		20	1.05524	23.19482	0.78658	19.25235
	30		20	1.06382	23.39367	0.77252	19.2254
	31		20	1.07712	23.51691	0.79584	20.15909
	32		20	1.08018	23.51037	0.79193	20.11131
	33		20	1.05228	23.29491	0.79362	19.4537
	34		20	1.06603	23.51691	0.797	19.98062
	35		20	1.05633	23.19398	0.78061	19.12529
	36		20	1.06519	23.40004	0.76659	19.10767
	37		20	1.08484	23.39619	0.73423	18.63544
	38		20	1.08598	23.31453	0.75111	19.01752
	39		20	1.09724	23.40457	0.77396	19.87567
	40		20	1.09607	23.40155	0.76031	19.50178

	41		20	1.09126	23.39971	0.77959	19.90689
	42		20	1.09077	23.40667	0.77987	19.911
	43		20	1.09983	23.52703	0.78003	20.18381
	44		20	1.07558	23.1872	0.76549	19.09118
	45		20	1.07169	23.4225	0.77602	19.47936
	46	108	19	1.05401	22.15365	0.78173	18.25354
	47		20	1.08849	23.08387	0.77464	19.46412
	48		20	1.11159	23.32961	0.74424	19.30031
	49		20	1.12082	22.68623	0.73014	18.56542
	50		19	1.11018	22.28226	0.75767	18.74262
	51		20	1.08525	23.40342	0.72427	18.39538
	52		20	1.08037	23.31495	0.77122	19.42628
	53		20	1.07715	23.00044	0.75604	18.73086
	54		20	1.05849	23.31383	0.77182	19.04661
	55		20	1.06206	23.45901	0.76891	19.15715
	56		20	1.12558	23.41994	0.75115	19.80092
	57		20	1.09271	23.52854	0.77245	19.85958
	58		20	1.10198	23.41868	0.7841	20.2352
	59		20	1.10447	23.63118	0.77188	20.14596
	60		20	1.11123	23.3136	0.74664	19.34295
	61		20	1.10264	23.52369	0.74644	19.36122
	62		20	1.10175	23.41774	0.7454	19.23175

	63		20	1.10433	23.42173	0.7383	19.09627
	64		20	1.10512	23.3147	0.73776	19.00881
	65		20	1.11222	22.59502	0.759	19.07423
	66		20	1.11123	23.3136	0.74664	19.34295
	67		20	1.1123	23.11076	0.74168	19.06581

Table S3: Comparison of perovskite module efficiencies reported on a different module size.

**References**	**Aperture Efficiency (%)**	**Active area efficiency (%)**	**Module area (cm^2)**	**Testing Organization / Test Method**
This work	19.60±3.60	20.67±3.80%	79.67	NREL/ Stabilized Eff
Science **2021**, *373* (6557), 902–907	19.15±0.23[1]		50.00	NREL/ Stabilized Eff
Prog. Photovoltaics Res. Appl. **2021**, *29* (7), 657–667		20.10±0.40[2]	63.98	JET/ Eff from J-V
Prog. Photovoltaics Res. Appl. **2021**, *29* (7), 657–667		17.90±0.50[2]	804.00	AIST/ Eff from J-V
Nat. Energy **2022**, *7* (6), 528–536.	15.30±0.00[3]		205.00	Not Certified
Science **2022**, *375* (6578), 302–306		20.60±0.00[4]	64.00	Not Certified
Nat. Photonics **2022**, *16* (2), 119–125		21.45±0.00[5]	25.00	Nano Convergence Practical Application Center/ Eff from J-V
Nat. Nanotechnol. **2022**, *17* (6), 598–605		22.72±0.00[6]	24.63	Photovoltaic and Wind Power Systems Quality Test Center/ Eff from J-V
Nat. Commun. **2021**, *12* (1), 6394		21.40±0.00[7]	26.00	Not Certified
Nat. Energy **2023**, *8*, 294–303	18.20±0.00[8]		17.10	National Institute of Metrology/ Stabilized Eff
Science. **2023**, *379* (6629), 288–294	19.60±0.00[9]		17.10	An Accredited Testing Laboratory/ Stabilized Efficiency
Science. **2021**, *372* (6548), 1327–1332		19.54±0.00[10]	65.00	Not Certified

Table S4: Effects of concentrations of $Zn(CF_3SO_2)_2$ on the photovoltaic parameters and series and shunt resistance for perovskite solar cells.

**PSCs**	**V_{OC} (V)**	**J_{SC} (mA/cm^2)**	**FF**	**PCE (%)**	**Shunt resistance (Ω cm^2)**	**Series resistance (Ω cm^2)**
**Control**	1.16	24.2	0.810	22.8	1474	3.13
**With 0.28% $Zn(CF_3SO_2)_2$**	1.18	23.8	0.850	23.9	1556	2.81
**With 0.42% $Zn(CF_3SO_2)_2$**	1.18	24.3	0.842	24.2	2010	2.74
**With 0.55% $Zn(CF_3SO_2)_2$**	1.19	24.2	0.829	23.9	2678	2.74

References

1. Chen, S. *et al.* Stabilizing perovskite-substrate interfaces for high-performance perovskite modules. *Science.* **373**, 902–907 (2021).
2. Green, M. A. *et al.* Solar cell efficiency tables (Version 58). *Prog. Photovoltaics Res. Appl.* **29**, 657–667 (2021).
3. Bu, T. *et al.* Modulating crystal growth of formamidinium–caesium perovskites for over 200 cm2 photovoltaic sub-modules. *Nat. Energy* **7**, 528–536 (2022).
4. Kim, M. *et al.* Conformal quantum dot-SnO2 layers as electron transporters for efficient perovskite solar cells. *Science.* **375**, 302–306 (2022).
5. Jeong, M. *et al.* Large-area perovskite solar cells employing spiro-Naph hole transport material. *Nat. Photonics* **16**, 119–125 (2022).
6. Ding, Y. *et al.* Single-crystalline TiO2 nanoparticles for stable and efficient perovskite modules. *Nat. Nanotechnol.* **17**, 598–605 (2022).
7. Liu, C. *et al.* Tuning structural isomers of phenylenediammonium to afford efficient and stable perovskite solar cells and modules. *Nat. Commun.* **12**, 6394 (2021).
8. Luo, L. *et al.* Stabilization of 3D/2D perovskite heterostructures via inhibition of ion diffusion by cross-linked polymers for solar cells with improved performance. *Nat. Energy* **8**, 294–303 (2023).
9. You, S. *et al.* Radical polymeric p-doping and grain modulation for stable, efficient perovskite solar modules. *Science.* **379**, 288–294 (2023).
10. Bu, T. *et al.* Lead halide-templated crystallization of methylamine-free perovskite for efficient photovoltaic modules. *Science.* **372**, 1327–1332 (2021).
