# Supplementary material for: Iodide manipulation using zinc additives for efficient perovskite solar minimodules
Source: Nat Commun. 2024 Feb 14;15:1355. doi: 10.1038/s41467-024-45649-6 (PMC10867015; doi:10.1038/s41467-024-45649-6)
Supplement: Supplementary file 3 — Solar Cells Reporting Summary [file 41467_2024_45649_MOESM3_ESM.pdf]

nature research

Corresponding author(s): Jinsong Huang

Solar Cells Reporting Summary

Nature Research wishes to improve the reproducibility of the work that we publish. This form is intended for publication with all accepted papers reporting the characterization of photovoltaic devices and provides structure for consistency and transparency in reporting. Some list items might not apply to an individual manuscript, but all fields must be completed for clarity.

For further information on Nature Research policies, including our data availability policy, see Authors & Referees.

Experimental design

Please check: are the following details reported in the manuscript?

1. Dimensions

Item	Yes/No	Details
Area of the tested solar cells	☒ Yes ☐ No	For single-junction perovskite devices, the working area was defined by a photomask with an aperture area of 8 mm2. For minimodule devices, the working area was defined by a photomask with an aperture area of 78, 84, and 108 cm2 determined by design and a calibrated imaging system (Methods).
Method used to determine the device area	☒ Yes ☐ No	We use the photomask and a calibrated imaging system to determine the device area (Methods).

2. Current-voltage characterization

Item	Yes/No	Details
Current density-voltage (J-V) plots in both forward and backward direction	☒ Yes ☐ No	Fig.1d, 2c, S2, and S6
Voltage scan conditions *For instance: scan direction, speed, dwell times*	☒ Yes ☐ No	Supplementary Information- Film and Device Characterization
Test environment *For instance: characterization temperature, in air or in glove box*	☒ Yes ☐ No	Supplementary Information- Film and Device Characterization
Protocol for preconditioning of the device before its characterization	☒ Yes ☐ No	No preconditioning is required before characterization.
Stability of the J-V characteristic *Verified with time evolution of the maximum power point or with the photocurrent at maximum power point; see ref. 7 for details.*	☒ Yes ☐ No	Fig.2e

3. Hysteresis or any other unusual behaviour

Item	Yes/No	Details
Description of the unusual behaviour observed during the characterization	☒ Yes ☐ No	Devices show negligible hysteresis behavior.
Related experimental data	☒ Yes ☐ No	We did not provide this data. But we will be available upon the request.

4. Efficiency

Item	Yes/No	Details
External quantum efficiency (EQE) or incident photons to current efficiency (IPCE)	☒ Yes ☐ No	Fig.S3
A comparison between the integrated response under the standard reference spectrum and the response measure under the simulator	☒ Yes ☐ No	Difference is negligible.
For tandem solar cells, the bias illumination and bias voltage used for each subcell	☐ Yes ☒ No	Not Applicable.

5. Calibration

Item	Yes/No	Details
Light source and reference cell or sensor used for the characterization	☒ Yes ☐ No	Supplementary Information- Film and Device Characterization

November 2017

Confirmation that the reference cell was calibrated and certified	☒ Yes ☐ No	Supplementary Information- Film and Device Characterization
Calculation of spectral mismatch between the reference cell and the devices under test	☐ Yes ☒ No	Calculation is not reported. But we recorded the spectra of light source and compared it with AM 1.5G spectra, as shown in Fig. S30 (Supplementary information).

6. Mask/aperture

Size of the mask/aperture used during testing	☒ Yes ☐ No	For single-junction perovskite devices, the working area was defined by a photomask with an aperture area of 8 mm2. For minimodule devices, the working area was defined by a photomask with an aperture area of 78, 84, and 108 cm2 determined by design and a calibrated imaging system (Methods).
Variation of the measured short-circuit current density with the mask/aperture area	☒ Yes ☐ No	The aperture area is fixed for each device. Negligible variation is observed.

7. Performance certification

Identity of the independent certification laboratory that confirmed the photovoltaic performance	☒ Yes ☐ No	Certification was performed by the National Renewable Energy Laboratory
A copy of any certificate(s)<br>*Provide in Supplementary Information*	☒ Yes ☐ No	Fig.2e

8. Statistics

Number of solar cells tested	☒ Yes ☐ No	Fig.1b and c, Fig.2d, Fig.S1, and Fig.S9
Statistical analysis of the device performance	☒ Yes ☐ No	Fig.b-c, Fig.2d, S1, and S6

9. Long-term stability analysis

Type of analysis, bias conditions and environmental conditions<br>*For instance: illumination type, temperature, atmosphere humidity, encapsulation method, preconditioning temperature*	☒ Yes ☐ No	Fig.1e, S4, and S6
